# Editorial Department

**Published:** 1869-02

**Authors:** 


Page(s) missing temperature it would destroy the tissues like red hot iron. The man in whose stomach this liquid would explode into gas, would certainly not be an object of envy. Surely such a paragraph as the above quotation is a most earnest practical appeal for the general diffusion of scientific instruction.

